# Syringomatous Adenoma of the Nipple in a Male Breast: A Case Report With a Brief Review of Literature and Histomorphological Approach to Diagnosis

**DOI:** 10.7759/cureus.19586

**Published:** 2021-11-15

**Authors:** Rajaguru Paramaguru, Subramaniam Ramkumar

**Affiliations:** 1 Pathology, Al Salam International Hospital, Kuwait City, KWT; 2 Pathology, PVS Memorial Hospital, Ernakulam, IND; 3 Pathology, Woodland Hospital, Shillong, IND

**Keywords:** syringomatous adenoma of the nipple, adenoid cystic carcinoma, sclerosing syringomatous carcinoma, adenosquamous carcinoma, tubular carcinoma, nipple adenoma, nipple retraction, male breast

## Abstract

Syringomatous adenoma of the nipple (SAN) is a benign and locally infiltrative lesion possibly arising from the sweat gland ducts in the nipple-areolar region. This rare lesion has been reported in the female breast; however, reports on the male breast are extremely rare. Although benign, SAN has a high risk of recurrence. The clinical presentation and histomorphological features often mimic a malignancy. Hence, an awareness of this lesion is required to make a correct diagnosis. In this report, we describe the histomorphological features of SAN in a male breast.

## Introduction

Syringomatous adenoma of the nipple (SAN) is a rare benign neoplasm that is locally infiltrative [1]. Because of its infiltrative behavior, SAN has a high risk of recurrence, which highlights the importance of recognizing this entity. However, metastasis has not yet been reported. SAN has a close resemblance to syringomatous tumors of the skin, suggesting a possible origin from the sweat gland ducts [1,2]. SAN is extremely rare in the male breast, with only a few case reports in the literature. In this report, we describe the histomorphological features of SAN in a 43-year-old male patient that clinically mimicked a malignancy.

## Case presentation

A 43-year-old male patient came to the surgery outpatient department (OPD) with complaints of a mass in the right breast for two years and pain over the mass for the past three months. On examination, there was a mass measuring 2 × 2 cm just beneath the nipple-areolar complex of the right breast. The mass was non-tender and firm in consistency. There was nipple retraction and no nipple discharge. A single axillary lymph node measuring 1 × 1 cm was palpable in the right axillary region. A mammogram was not performed.

The patient was advised fine needle aspiration cytology (FNAC). Fine needle aspiration (FNA) was performed from the right breast lesion, and the smears showed numerous cohesive clusters of ductal epithelial cells with fine chromatin, inconspicuous nucleoli, and moderate cytoplasm. Few stromal fragments and naked nuclei were also noted. Atypical cells were absent. Hence, a diagnosis of proliferative breast lesion without atypia was given. FNA was also performed from the axillary lymph node, which showed scattered reactive lymphoid cells. Atypical cells were absent.

**Figure 1:**
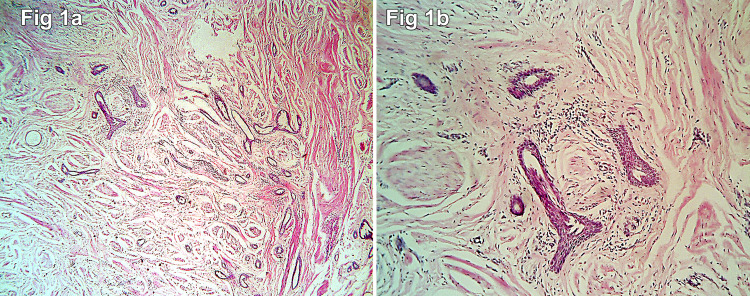
(a) Tissue section showing small- and medium-sized elongated tubules infiltrating the smooth muscle fibers. Focal squamous metaplasias of the tubules are seen (H&E 100×). (b) Tissue sections showing squamous cysts filled with keratin flakes (H&E 40×).

**Figure 2:**
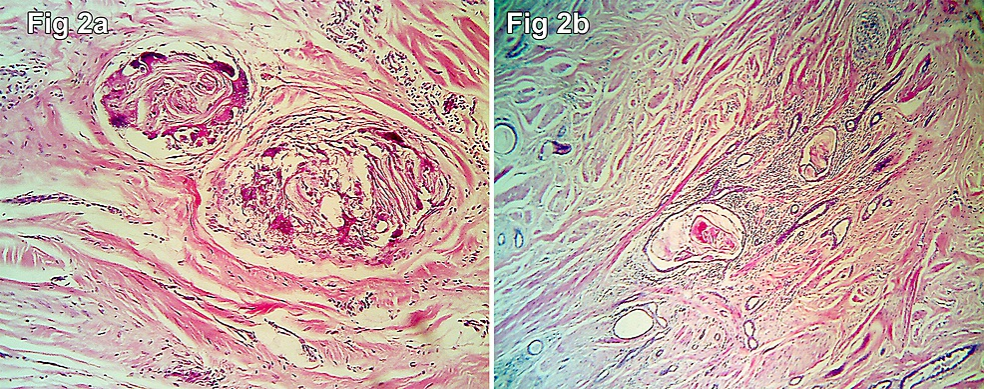
Tissue sections showing foreign body giant cell reaction to the ruptured squamous cyst (a: H&E 40×; b: H&E 100×).

Subsequently, wide local excision of the lesion was performed, and the specimen was sent for histopathological examination. The specimen measured 6 × 4 × 2 cm and was a skin-covered fibrofatty tissue. The overlying nipple showed retraction, and the areola appeared firm on palpation. On slicing, there was an ill-defined grey-white lesion in the retroareolar region measuring 2 × 2 × 1 cm. The resection margins were grossly free of tumor. Microscopic examination revealed an ill-circumscribed and infiltrative lesion composed of small- to medium-sized irregular comma-shaped ducts lined by double-layered epithelium. Focal squamous cysts with evidence of rupture in the form of foreign body giant cell reaction and focal calcification were seen (Figure 1 and Figure 2).

**Figure 3:**
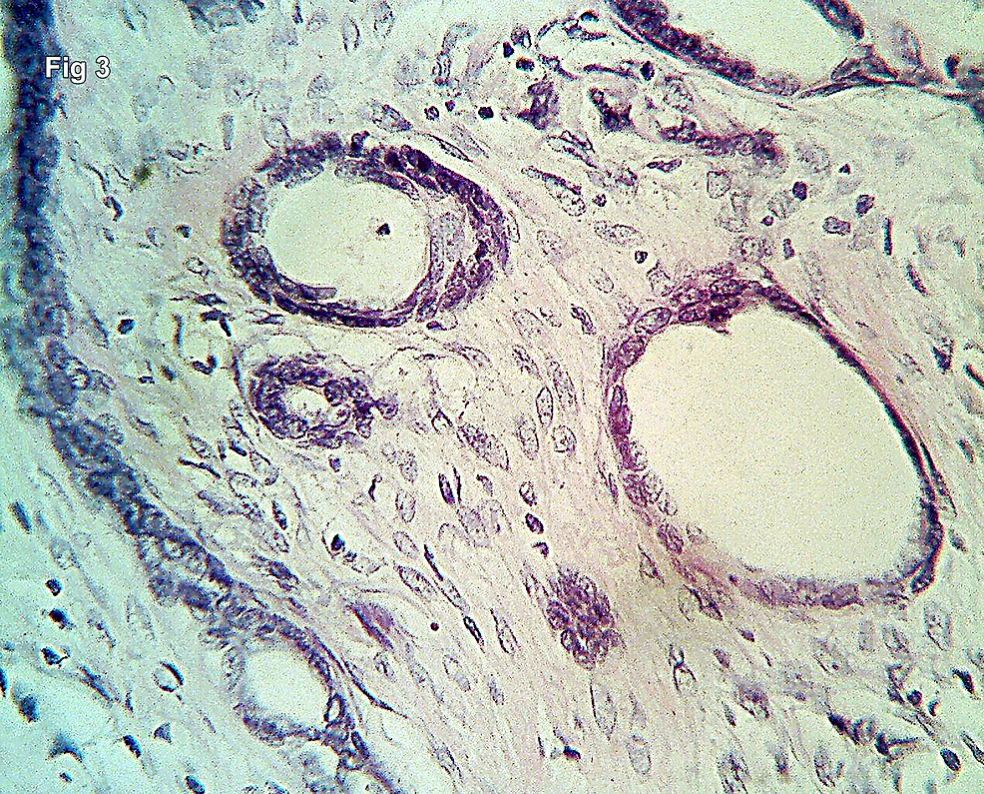
High-power view showing the fibromyxoid nature of the stroma and the dual lining epithelium of the ducts (H&E 400×).

Few of the ducts appeared elongated. The ducts were embedded in a fibromyxoid stroma (Figure 3).

**Figure 4:**
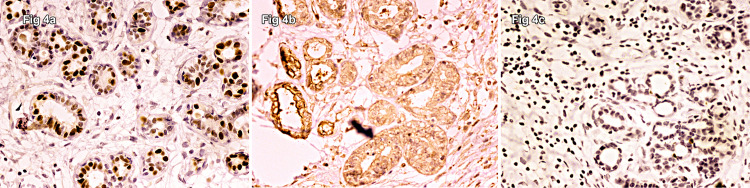
Neoplastic cells showing strong nuclear immunostaining for (a) ER, (b) PR, and (c) CD15.

These ducts were also seen to infiltrate the smooth muscles and around the nerve bundles. The surgical margins were uninvolved microscopically as well. The neoplastic cells showed strong nuclear immunostaining for ER, PR, and CD15 (Figure 4).

**Figure 5:**
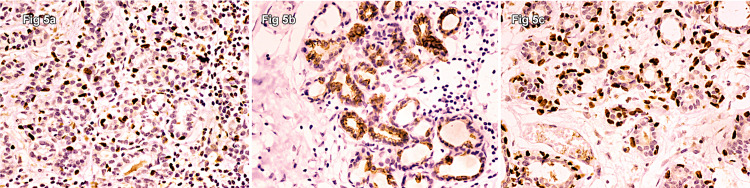
Immunostains for myoepithelial/basal cell markers, including (a) smooth muscle myosin, (b) p63, and (c) cytokeratin 5/6 revealed an intact myoepithelial layer surrounding the tubules and nests, highlighting the noninvasive nature of this infiltrative lesion.

The immunostains for myoepithelial/basal cell markers, including smooth muscle myosin, p63, and cytokeratin 5/6 (CK5/6) (Figure 5), revealed an intact myoepithelial layer surrounding the tubules and nests, highlighting the noninvasive nature of this infiltrative lesion.

Table 1 describes the antibodies used in the study, as well as their respective titers.

**Table 1:** Immunohistochemical markers with respective antibodies and titers used in the study.

No.	Name of the antibody	Source	Clone	Dilution	Name of the supplier
1	CK 5/6	Rabbit monoclonal	EP24/Ep67	1:50–1:100	PathnSitu, Livermore, CA, USA
2	P63	Mouse monoclonal	4A4	1:50–1:00	PathnSitu, Livermore, CA, USA
3	CD15	Rabbit monoclonal	EP273	1:50–1:100	PathnSitu, Livermore, CA, USA
4	ER	Rabbit monoclonal	Ep1	1:25–1:50	PathnSitu, Livermore, CA, USA
5	PR	Rabbit monoclonal	EP2	1:100	PathnSitu, Livermore, CA, USA
6	GCDFP-15	Rabbit monoclonal	EP95	1:100	PathnSitu, Livermore, CA, USA
7	Cytokeratin high-molecular-weight (HMW) CK2	Mouse monoclonal	34BE12	1:100	PathnSitu, Livermore, CA, USA

A diagnosis of benign syringomatous adenoma with complete excision in the plane of the lesion was made. The patient was asymptomatic with no recurrence on follow-up.

## Discussion

The differentiation of sweat ducts can be observed in syringomatous adenoma of the nipples (SAN) [1]. Moreover, such differentiation can be described as a non-metastasizing, locally invasive tumor of the nipple or areolar region. Jones initially described nipple adenoma (NA) in 1955 as an exceptionally rare, benign nipple tumor [2,3]. In a preliminary investigation of five patients in 1983, Rosen defined SAN as a distinct diagnostic entity [4].

According to our information, in medical literature, only 38 instances of SAN have been documented, including 36 female cases and two male cases [5-9]. Such patients varied in age from 11 to 87 years old, with a mean age of 46.1 years at presentation [9]. The size range of the removed tumors varies from 5 to 40 mm in diameter, with an average of 17.7 mm [9]. On two occasions, bilaterality was established [10,11]. Only a few occurrences of SAN have been reported in males or babies, as SAN usually appears in women in their fourth or fifth decades of life [1]. Of children with SAN who were investigated, most were girls, except for one boy and an infant [1].

The specific anatomical origins of this lesion are unknown [2]. The lack of epithelial growth in the mammary ducts and the absence of a link to the epidermis in most instances indicate an unknown cause. Because random portions of nipples obtained from breasts excised for mammary cancer occasionally show sweat gland ducts, SANs can be considered to be developing from these appendages [3]. As a result, SANs are thought to be morphologically identical to syringomas, which are eccrine duct tumors. Another theory is that SANs develop from pluripotent adnexal keratinocytes that differentiate into both follicles and sweat glands [12].

Morphologically, SAN presents as an isolated hard lump in the subareolar or nipple area of the unilateral breast. However, two cases have been reported with a gross presentation of an erosive fungating appearance [4,11]. Bilateral involvement has also been documented in the past [5,12]. Pain, soreness, itching, crusting, ulceration, and nipple discharge or inversion are other possible modes of presentation [13].

Mammography findings

SAN is characterized by a thick and irregularly shaped mass in the subareolar area with spiculated microcalcification in foci. An unclear heterogeneous mass with inner echoes can be detected by ultrasonography. Mammography (MMG) cannot detect the mass, whereas magnetic resonance imaging (MRI) can. Moreover, the imaging results of SANs are similar to those of malignant tumors; therefore, MMG, ultrasonography, and MRI may be ineffective techniques for distinguishing SAN from carcinoma [14]. Although fine needle aspiration or needle biopsy is typically insufficient to accurately diagnose SAN, these procedures typically record many instances as being suspicious of malignancy.

Macroscopy

SAN can range from 1 to 3 cm in diameter. The cut surface reveals an ill-defined tumor with robust firmness and is hard. The cut surface is uniformly grey or white with cystic areas. The areolar skin and breast tissue underneath are usually unremarkable [4,9,12].

Microscopy

The epidermis may exhibit different acanthosis and pseudoepitheliomatous hyperplasia on histological examination. The infiltrating pattern of SAN is defined by clusters and twisting cords of cells creating glandular formations within the stroma (Figure 1a and 1b). Perineural invasion is common [14,15]. Near the skin’s surface, small keratinous cysts form. Two cell layers covering these ducts, which have a teardrop, tadpole, or comma shape, create the appearance of a syringoma. Between the fibrous stroma and epithelial cells, a separate layer of myoepithelial cells might be observed at higher magnification (Figure 1c and 1d). It is possible that the underlying breast tissue is normal or has hyperplastic alterations. The overlying skin or nipple epidermis involvement is not linked with SAN.

Diagnostic criteria

The morphological and histological criteria for a SAN diagnosis are lesion in the nipple or areola localized in dermis and subcutis; compressed, comma-shaped, irregular tubes infiltrating into smooth muscle bundles and nerves; tubules surrounded by myoepithelial cells; cysts bordered with stratified squamous epithelium and filled with keratinous material; and absent necrosis and mitotic activity.

Differential diagnoses

The following lesions should be considered in the differential diagnosis of SAN.

Nipple Adenoma (NA)

Histologically, a SAN is identical to a syringoma, which is a benign tumor that arises in the dermal sweat gland ducts; the latter has clinical characteristics that differentiate it from SAN, such as presenting as a single nipple lesion [12].

NA causes serous or bloody nipple discharge and is frequently confused with syringomatous adenoma (SA). NA shows epithelial hyperplasia with homogeneous myoepithelial cells under microscopy. A lactiferous duct displacing the nipple stroma causes growth. In contrast to NA, which is a confined lesion, SAN has a stromal infiltrate. Both SA and NA may have squamous metaplasia [16].

Florid papillomatosis (FP) is a primarily hyperplastic epithelial proliferation of the major lactiferous ducts that might mirror SA but without squamous cysts or other histological characteristics. In contrast with SA, patients with FP are older and are more likely to suffer nipple erosion with bleeding with a relatively short duration [16].

Tubular Carcinoma (TC)

SAN is frequently misdiagnosed as TC of the breast. The site of TC is normally in the upper outer part of the breast or far from the nipple; it can also appear in the subareolar area or nipple, with an infiltrative growth that is difficult to differentiate from SA [16]. TC might also induce nipple retraction or Paget’s disease if it spreads to the nipple [9,15]. Teardrop or comma-shaped tubules of SAN are comparable to angular, pointed glands of TC, and both TC and SAN penetrate smooth muscle and nearby nerves [16].

Over 90% of tubules in TC have low-grade characteristics and open lumina with apocrine-like snouts and basophilic secretions, whereas SA has a compressed lumina (Figure 1b). Paget’s disease and micropapillary or cribriform forms of low-grade ductal carcinoma in situ are linked to TCs. TC also lacks a basement membrane and myoepithelial cells [16]. In challenging situations, an immunohistochemical stain for p63 and/or smooth muscle myosin heavy chain might be beneficial (Figure 5). The existence of squamous metaplasia, which is not a characteristic of TC, promotes SA diagnosis [16].

Adenosquamous Carcinoma

Low-grade breast adenosquamous carcinoma (ASC), which is a well-differentiated tumor with both glandular and squamous differentiation, might be mistaken for SAN. Under low magnification, it is indicated as compressed lumens with infiltrating visible growth patterns. Both tumors can recur and infiltrate deeply; some researchers use the term “SA [rg5]” interchangeably with “low-grade adenosquamous carcinoma” [16].

When it comes to low-grade adenosquamous carcinoma, differential diagnosis is not always easy to make. Therefore, we believe and agree with Rosen and others, who asserts unequivocally that despite certain anatomical similarities, the neoplastic processes of the two lesions are not the same [4-16]. Low-grade ASC of the breast is a low-grade form of metaplastic carcinoma of the breast with signs of metastasis [15], whereas SA of the nipple is benign and does not metastasize. The breast parenchyma is where low-grade ASC begins and spreads to the skin. The presence of pleomorphism and nuclear hyperchromasia can always be detected [16].

SA, on the other hand, develops from the skin; the basal parenchyma is only superficially involved, and nuclei are benign [16]. Smooth muscle actin staining reveals a second layer of myoepithelial cells, which indicates that the lesion is not a carcinoma. TC can be distinguished from SA and low-grade ASC on the basis of the hormone receptor status. SA and ASC are generally estrogen negative [16], whereas TC is estrogen positive.

Sclerosing Syringomatous Carcinoma

The infiltrating ducts of SAN demonstrate a broad-based and horizontal style of infiltration, in contrast to sclerosing syringomatous carcinoma, which is characterized by deep, vertical stromal infiltration and significant perineural dissemination [6].

Adenoid Cystic Carcinoma

Infiltrating SAN can be differentiated from other malignant breast neoplasms based on immunohistochemistry, which shows epithelial cell proliferation and significant stromal and basement membrane components in adenoid cystic carcinoma [6,7].

The occurrence of a distinct myoepithelial cell component in these tumors is debatable due to a limitation of comprehensive immunohistochemistry investigations of these rare tumors [11,12,17].

Multiple myoepithelial/basal cell indicators, such as smooth muscle myosin (SMM), p63, and basal cytokeratin 5/6, were strongly and diffusely immunoreactive in outer cell layers surrounding invading tumor nests and tubules in most instances (Table 2). Because few breast carcinomas express p63 or high-molecular-weight cytokeratins (HMW CK), the immunoreactivity values of the subpopulations of luminal epithelial cells for p63, high-molecular-weight cytokeratin 34E12, and cytokeratin 5/6 were consistent with a tumor of sweat duct origin. Low-grade ASC and SAN have histological and immunophenotypic similarities, although the former is differentiated by its placement in the peripheral deeper breast, with only uncommon nipple participation [11,12,17].

**Table 2:** Immunohistochemical profile of syringomatous adenomas.

IHC marker	Marker staining pattern in myoepithelial and epithelial cells
SMM	Diffusely and strongly positive in the outer myoepithelial cells of tubules and nests; negative in luminal epithelial cells
HMW CK	Diffusely and strongly positive in both myoepithelial cells and luminal epithelial cells
CK5/6	Diffusely and strongly positive in myoepithelial cells; heterogeneously moderately to strongly positive in epithelial cells
p63	Diffusely positive in nuclei of myoepithelial cells; positive in the nuclei of epithelial cells with squamoid differentiation
CD15	Predominantly negative with focal heterogeneous positivity of epithelial cells
GCDFP-15	Predominantly negative with focal weak positivity of epithelial cells
ER	Weakly to moderately positive in the nuclei of 10% of epithelial cells
PR	Negative

Treatment and prognosis

Moderate but highly destructive SAN is frequently misinterpreted as tubular carcinoma. The unique clinical manifestations and mammographic results of SAN might lead to an erroneous diagnosis of “very suggestive of malignancy.” Therefore, pathologists and clinicians should pay careful attention to this benign lesion to avoid misdiagnosis and needless surgery [11,16]. Seven of the 11 patients in the study of Jones et al. had already been classified as well-differentiated TC or were referred for consultation to rule out this possibility [15].

A small needle aspiration interpreted as likely low-grade adenosquamous carcinoma led to mastectomy with axillary dissection in the case described by Toyoshima et al. [18]. Subsequently, SAN was discovered in continuous histological examination. In a case report published recently by Alhayo et al., the core biopsy of a nipple ulcer in a 53-year-old female was initially classified as ductal cancer [19], but subsequent wedge biopsy revealed it to be NA [20]. As described in a single instance, frozen sections can be mistaken as a malignant tumor, thus ending in a mastectomy [20].

In situations when SAN is detected in the dermis and subcutis of the nipple or areola [4], appropriate treatment requires complete excision of the nipple-areolar complex with histologically negative margins [9,18]. SAN is not a cancerous tumor, and there have been no instances of it spreading to other parts of the body [5]. A solitary incidence of suspected micrometastasis from such a tumor to a sentinel lymph node has been documented [6,21].

Although with appropriate informed consent, if a patient desires nipple preservation, tumor excision with nipple restoration should be offered. Resection of the nipple can produce significant aesthetic effects. Reconstruction following central mound excision has recently become a viable treatment option [14]. If the tumor is so close to the nipple that nipple preservation is difficult, the patient should be given an effective treatment. However, in such instances, thorough postoperative surveillance is required.

Throughout a follow-up phase of one to six years, there was no indication of relapse in patients who had negative margins following excision of a complete SAN. Patients with positive margins following local surgical removal, on the other hand, had tumor recurrence. Recurrence times were found by Jones et al. to range from 1.5 months to 4 years [15]. As a result, if the follow-up was comprehensive, it should last longer than five years [15]. As a result, vigilant supervision is required to detect local recurrence [14]. Local re-excision was used to treat the majority of recurrences. The metastasis of SAN was unknown if removed incompletely, despite its local aggressiveness and recurrence [14].

## Conclusions

SAN is a benign neoplasm of the breast that necessitates a proper histomorphological diagnostic approach with a high index of suspicion. The practicing pathologist should be aware of this increasingly diagnosed yet uncommon entity so that an erroneous diagnosis does not result in an unnecessary radical procedure.
